# Overall Accuracy of Radiological Digital Planning for Total Hip Arthroplasty in a Specialized Orthopaedics Hospital

**DOI:** 10.3390/jcm12134503

**Published:** 2023-07-05

**Authors:** Serban Dragosloveanu, Mihnea-Alexandru Petre, Mihai Emanuel Gherghe, Dana-Georgiana Nedelea, Cristian Scheau, Romica Cergan

**Affiliations:** 1 Department of Orthopaedics and Traumatology, The “Carol Davila” University of Medicine and Pharmacy, 050474 Bucharest, Romania; 2 Department of Orthopaedics, “Foisor” Clinical Hospital of Orthopaedics, Traumatology and Osteoarticular TB, 021382 Bucharest, Romania; 3 Department of Physiology, The “Carol Davila” University of Medicine and Pharmacy, 050474 Bucharest, Romania; 4 Department of Anatomy, The “Carol Davila” University of Medicine and Pharmacy, 050474 Bucharest, Romania; 5 Department of Radiology and Medical Imaging, “Foisor” Clinical Hospital of Orthopaedics, Traumatology and Osteoarticular TB, 021382 Bucharest, Romania

**Keywords:** total hip arthroplasty, digital planning, digital templating, radiological planning, radiology

## Abstract

Preoperative radiological planning is a key factor in the prediction of implant size and positioning that influences surgical time, the risk of complications, and functional outcomes. We have tested the accuracy of the digital templating performed in our hospital for a sample of 215 patients that underwent total hip arthroplasty. We assessed the accuracy of correctly predicting implant size for the femoral and acetabular components, as well as the stem neck length. We found that our method of templating proved accurate (within one size) in 95.8% of cases for the stem and 94.9% for the cup when using the anteroposterior view only, while the lateral view was accurate in 95.8% of cases for the stem and 97.2% for the cup. Exact prediction of the stem size was obtained in 77.7% of cases using the anteroposterior view and 67.0% of cases on the lateral view, and 73.0% and 74.4% of cases for the cup on the AP and LL views, respectively. Stem neck size was predicted exactly in 75.35% of cases and within one size in 93.49% of cases. We concluded that our method of digital templating using dedicated software is highly effective in accurately predicting implant size.

## 1. Introduction

Arthroplasty is a reliable and increasingly used procedure that returns patient mobility and improves their quality of life [1]. The majority of procedures are applied to the hip and knee joints, and the surgical methods, perioperative care, and outcomes are constantly improving [2,3].

One of the key factors in total hip arthroplasty is the adequate preoperative assessment of the patient and determining the optimal size, type, and position of the implant components. Accurate planning of implant size is an important factor in reducing surgical time and limiting potential perioperative complications, as well as predicting correct implant positioning, alignment, and leg length [4,5,6]. Currently, various templating methods are employed in orthopedic services worldwide. Plain physical X-ray films are still in use in some centers, while most orthopedic surgeons have transitioned to using digital images [7,8]. Using images displayed on monitors, planning may be performed manually or computer-assisted, and either software plugins or acetate films may be used to overlay the implant on the patient’s preoperative image [9,10]. Radiological planning may involve two- or three-dimensional templating, which may be performed using plain X-rays or computed tomography (CT) [11,12].

There is an ongoing controversy regarding the best method for preoperative planning in patients with total hip arthroplasty (THA). Multiple parameters are to be considered, such as cost-efficiency, availability, implant type, surgeon experience, and many more. Recent literature studies cite that the accuracy of correctly predicting implant size (within one size) is 86 to 100% for the acetabular cup and 94 to 100% for the femoral stem when using 3D planning [13,14,15]. Some studies report that CT is better than 2D planning in predicting acetabular cup size while stating that acetate and digital templating yield similar results, both overpredicting implant size [16,17]. However, some authors have shown that 3D CT and digital templating have similar results in calculating some landmarks for planning THA [18]. Furthermore, there are reports that acetate templating on digital images has better results than digital planning in THA templating [19].

Accurate preoperative planning is of great significance for a successful THA. Proper planning increases the precision of the procedure, enables the surgeon to anticipate potential difficulties, such as leg length discrepancies or periprosthetic fractures, and helps the surgeon to reproduce hip biomechanics while shortening the overall duration of the surgery [20,21,22].

Our study aimed to assess the accuracy of the radiological digital planning method used in our dedicated orthopedics hospital in patients with THA.

## 2. Materials and Methods

### 2.1. Study Design

Our retrospective study included 215 consecutive patients that underwent THA between July 2021 and March 2023 in “Foisor” Clinical Hospital of Orthopaedics, Traumatology, and Ostheoarticular TB. The study aimed to determine the accuracy of digital templating in estimating the correct size of implants. All patients in our study received Zimmer Uncemented Metabloc Stem and corresponding Zimmer Trilogy Multi-holed/Non-holed acetabular shell prosthetic implants, and the radiological digital planning and subsequent surgeries were performed by the same team. Patients with hip dysplasia, septic osteoarthritis, or femoral neck fracture were excluded from the study, as were patients that underwent revision THA. We used the hospital management software to identify and include the patients that met the following inclusion criteria: hemoglobin levels >12 mg/dL for men and >11 mg/dL for women, glycated hemoglobin <7.5%, no urinary symptomatology and negative urine culture test, C-reactive protein <1.5 mg/dL with the exception of rheumatological or oncological conditions, and white blood cell count <11,000 cells/µL.

The study was approved by the Ethics Committee of “Foisor” Clinical Hospital of Orthopaedics, Traumatology, and Ostheoarticular TB (registration no. 4702/15.05.2023). Written informed consent was obtained from all patients. The study followed the ethical principles for medical research stipulated by the Declaration of Helsinki in 1964 and its later amendments.

### 2.2. Image Acquisition and Analysis

All patients undergoing THA are routinely submitted to anteroposterior (AP) and lateral (LL) hip radiographs in our Department of Radiology and Medical Imaging. All X-ray investigations were performed on a DigitalDiagnost R3.1 machine (Philips Medical Systems Nederland B.V, Amsterdam, The Netherlands). The anteroposterior view is performed with the patient supine, with arms alongside the body, extended lower limbs, with heels at 10 cm apart and halluces in contact. The central beam is vertical, perpendicular to the table, and centered midway between the umbilicus and pubic symphysis. The lateral view is obtained with the patient in lateral decubitus, rolled forward at 15°. The examined thigh is slightly flexed, and the opposite thigh is flexed up to 90° with the knee placed on a sandbag. The central beam is vertical and enters the gluteal region, exiting through the greater trochanter (of the examined side). Both views are performed with an antidiffusion X-ray grid and a focus-film distance of 115 cm. Exposure parameters are 77 kV and 160 mA. The digital images are verified for quality and then archived.

For each patient, we analyzed the radiological images saved in the hospital picture archiving and communication system (PACS). For all patients, a dedicated workstation connected to the hospital PACS was used. The imaging software Cedara I-View 6.3.2 (Ontario, Canada) and dedicated orthopedics modules were used for the radiological planning of the stems and cups. The preoperative anteroposterior and lateral hip X-rays of all patients were examined. The hip osteonecrosis score, according to the Steinberg classification, was also recorded for each patient [23]. Further morphological measurements were performed, including the limb-length discrepancy and the femoral medial offset, as presented below.

### 2.3. Digital Templating

**Figure 1:**
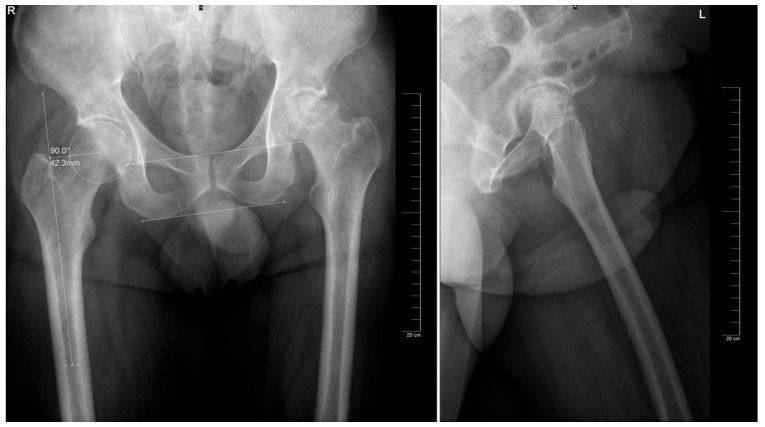
Identification of relevant landmarks and measurements of the hip and on the non-target side, on the anteroposterior and lateral view, respectively (bi-ischial line, interteardrop line, femoral offset).

**Figure 2:**
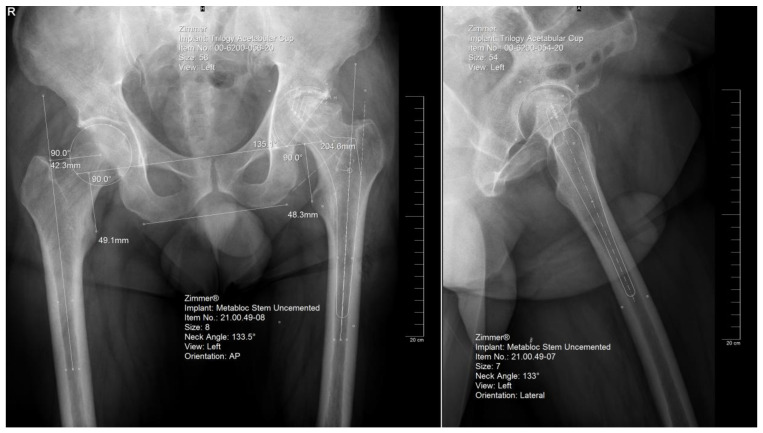
Templating of the femoral and acetabular components on the anteroposterior and lateral view, respectively. Note that there is one size difference in the estimation for each component between plans on the two views.

**Figure 3:**
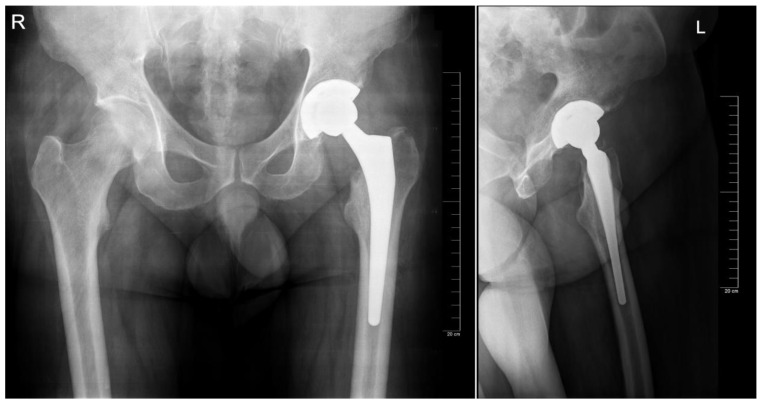
Postoperative aspect of the implant on the anteroposterior and lateral view, respectively. Note: actual implant sizes were size 8 for the femoral component and size 56 for the acetabular component.

The preoperative planning was performed manually in several phases. Starting with the anteroposterior view, the X-ray magnification is determined. Next, the landmarks are identified: the center of the femoral head, teardrop, anatomical axis of the non-target femur, and the ilioischial and transacetabular teardrop lines (Figure 1). The acetabular component size that fits the contour of the acetabulum was selected. The medial border is defined as the teardrop, and the cup is placed at 45 degrees of abduction. Afterward, the femoral component size that fits most precisely the contour of the canal is chosen, keeping in mind that it should be in contact with the lateral and medial cortex and must be in line with the anatomical axis of the femur. The upper part of the stem component must be located in the piriform fossa. The leg length discrepancy (LLD) is determined by the perpendicular distance from the middle of the lesser trochanter to the horizontal line drawn through the base of both teardrops, and the femoral medial offset (FMO) is calculated as the distance from the center of the femoral head perpendicular to the anatomical axis of the femur. The planning is performed on the lateral view using a similar approach (Figure 2). The acetabular template cup of appropriate size is placed with approximately 45 degrees of abduction. The medial border of the acetabular cup is positioned next to the ilioischial line and in close contact with the teardrop. The anatomical axis of the femur is marked, and the suitable femoral stem is chosen and positioned to fill the medullary canal. Postoperative radiographs are routinely performed to confirm proper positioning (Figure 3).

The digital planning in our study was performed by two trained orthopedic surgeons with experience in more than 1000 previous preoperative plans, and the findings were validated by a senior orthopedic surgeon that performed the surgical procedure. A perfect match between the planned and implanted components was labeled as “exact.” An exact match or variance within of size (plus or minus) was considered an accurate prediction.

### 2.4. Surgical Procedure

All patients who took part in this study underwent the same surgical procedure. The THA was performed using the direct anterior (Smith-Peterson) approach performed by the same surgeon [24]. This approach utilized the intervals between the sartorius and tensor fascia lata muscle in the superficial plane. Capsulotomy and hip dislocation allowed for the femoral head to be removed. The acetabulum was prepared using multiple reamers until adequate bone coverage of the cup was achieved. Preparation of the femoral canal was performed using increasingly larger diameter rasps until proper contact between the stem and cortices was sufficient. Trial components were positioned, and the reduction of hip prostheses was performed. A rigorous range of movement exams was performed to assert the stabilization of the hip prosthesis.

### 2.5. Statistical Analysis

The study data were analyzed using SPSS software version 23 (IBM, Armonk, NY, USA) and MedCalc^®^ Version 14.8.1 (MedCalc Software bvba, Ostend, Belgium). Data distribution was tested for normality using the Shapiro–Wilk test. Comparison of ages between groups was performed using a *t*-test assuming unequal variances, two-tailed. The association between categorical variables was assessed using a chi-square test. Results are presented as mean (standard deviation, SD) where appropriate. Statistical significance was considered when *p* values < 0.05.

## 3. Results

Our study comprised 215 patients (108 male and 107 female) aged between 33 and 86 years old, with a median age of 66. Patient body mass index (BMI) ranged between 19.6 and 43.3 kg/m^2^ with an average of 29.03 kg/m^2^. The presence and degree of avascular necrosis were also assessed in the study population, as were other radiological parameters. All relevant patient demographics are available in Table 1.

Regarding the predicted sizes for the stem and acetabular components, the results are listed in Table 2. The accuracy for predicting the stem dimension within one size (exact match, one size smaller, or one size larger) using the AP or LL images was 95.81%, while the accuracy for predicting the cup dimension within one size was 94.88% when using the AP projection and 97.21% when using the LL images. Stem neck size was predicted exactly in 75.35% of cases and within one size in 93.49% of cases.

Subsequently, we assessed the agreement between the AP and LL radiographs. The results are presented in Table 3.

We verified whether BMI influenced the prediction rates of a perfect match and found the following statistical significance of the differences between perfect match and mismatch regarding BMI: for the stem on AP view: *p* = 0.384 and LL view: *p* = 0.053, and for the cup on AP view: *p* = 0.409 and LL view: 0.080.

The presence or absence of AVN did not correlate with an exact prediction for the stem on AP (*p* = 0.537) or LL view (*p* = 0.332) nor for the cup on AP (*p* = 0.234) or LL view (*p* = 0.708).

Moreover, no influence of radiologically assessed leg length discrepancy or femoral medial offset was noted on the accuracy of predicting the correct sizes of the femoral or acetabular components. For LLD, the statistical significance of the differences between perfect match and mismatch for the stem, on AP and LL views, as well as for the cup, on AP and LL views, were: *p* = 0.716, *p* = 0.234, *p* = 0.298, *p* = 0.184 in the respective order. For FMO, in the same order, we obtained the following values: *p* = 0.574, *p* = 0.527, *p* = 0.831, *p* = 0.250.

However, there was a significant difference in the ages of the patients, specifically patients where stem size matched the digital templating were older compared to those where sizes were predicted to be either larger or smaller, and the correlation was found both on the AP projection (65.16 ± 9.67 years old vs. 61.88 ± 10.99 years old, *p* = 0.0455) and on the LL projection (65.69 ± 9.37 years old vs. 62.08 ± 11.17 years old, *p* = 0.0387). For the acetabular component, age did not play a role in the accuracy of the prediction.

## 4. Discussion

Previous literature reports have shown that a good prediction of implant size helps prevent surgical complications and shortens operative time while optimizing the performance of the implant in terms of restoring the range of motion and functional mobility of the patient [25,26].

We obtained an accurate prediction of implant size in 95.8% of cases for the stem for both AP and LL views. Regarding the cup, the prediction of the implant dimension within one size was 94.9% to 97.2% using either the anteroposterior or the lateral views, respectively. Exact prediction of the stem size was obtained in 77.7% of cases using the anteroposterior view and 67.0% of cases on the lateral view, and 73.0% and 74.4% of cases for the cup on the AP and LL views, respectively. These findings are similar to other literature reports using digital templating, which have obtained around 90% accuracy in predicting the prosthetic number within one size, and the values are generally higher than most other results [11,27,28,29,30].

Stem neck size was accurately predicted (within one size) in 93.49% of cases and exactly predicted in 75.35% of cases. This is similar to reports from another study; however, literature data on the prediction of stem neck size are scarce [31].

The added value of using the lateral view in the prediction of implant size was further evaluated, considering that most studies either use the frontal or lateral view [32,33,34]. We obtained a complete agreement between projections in 58.6% of cases for the stem and 60.0% of cases for the cup. We found that the lateral view mostly underestimated the implant size, while the AP view mostly overestimated it.

As previously mentioned, there is a wide variety of methods for THA preoperative planning that yield various results. The use of intraoperative radiological images could be useful in some cases where complications arise; however, a surgeon with good experience in the operating theater working with good quality planning makes their use unnecessary [35]. Supplementary radiographic projections such as the alar oblique and obturator oblique may yield additional data on the acetabular component. However, our software is not equipped nor validated for these projections; therefore, we did not consider them in our study [36]. CT is increasingly used in recent studies for planning hip arthroplasty due to its significant added advantage in providing 3D data and was shown to be superior in accuracy in predicting implant size as well as implant alignment [37]. Furthermore, eliminating the human factor as a source of bias was also attempted and successfully achieved in planning the acetabular cup size. A recent paper showed that building an atlas of a pelvis-cup merged statistical shape model and another for the statistical residual thickness map can be used to automatically plan the cup size with errors similar to manual segmentation performed by an experienced surgeon [38]. Moreover, new software can generate 3D models based on plain weight-bearing X-rays, which can be used to obtain better predictions of implant sizes compared to conventional 2D radiographs and equal to those obtained on CT, but in weight-bearing and with lower radiation doses to the patient [39]. The abovementioned methods can contribute to the decrease of the overestimation and underestimation in digital templating based on AP and LL views.

We considered various demographic and disease-related factors that might influence accurate, correct implant sizes prediction, such as gender, BMI, the presence and degree of AVN, or patient LLD of femoral medial offset. While these factors have been implicated in various literature reports, none had a statistically significant impact on our study [40,41,42].

However, we identified a statistically significant role of age as a predictor of good fit in our patient group. Patients with exact match predictions were significantly older than patients where implant size was either over- or underpredicted, and this was true for using the frontal or lateral views. Various studies have developed prediction models using age and other demographics to estimate the correct implant size; however, this was beyond the scope of our paper [40,43,44]. The templating software could be further refined, and this would be of great use, especially if it can account for demographic parameters of the population, such as age, gender, race, BMI, or other factors that were demonstrated to play a role in predicting implant size. Moreover, using deep learning artificial intelligence algorithms was shown to be a fast and accurate method [45,46].

In our study, the planning was performed by highly trained orthopedic surgeons. The experience of the planner does not seem to be a relevant factor in correctly estimating the implant size, as shown by other papers [47,48,49]. However, various reports suggest that residents or debutant surgeons may have lower accuracy in predicting the correct sizes for the THA implants, especially of the femoral component [34,50].

Our study was performed exclusively in one orthopedic hospital, which represents a study limitation. Furthermore, our hospital is a dedicated orthopedic hospital performing more than 1500 THAs per year. This may play an important role in the performance of digital planning and should be taken into consideration when comparing data from other centers. Additionally, our study tested solely one specific software and method of planning, and the templating and surgery were performed by one surgical team.

## 5. Conclusions

Digital templating using dedicated software is an inexpensive, low-risk, widely available, and highly effective method in accurately predicting implant size and shows performance comparable to other newer methods, such as 3D or CT-based templating. We found no impact of patient demographics on the accuracy of implant size prediction, except for age. AVN, LLD, or femoral offset did not influence the prediction. Using complementary radiographic projections may prove useful in improving prediction accuracy. We noted, however, that in the lack of consensus, the lateral view underestimated the implant size while the frontal view overestimated it. Further studies are required to assess the definitive role and the best approach of radiological digital planning for total hip arthroplasty, considering the variability in method, technique, and implant model in literature reports.

## Figures and Tables

**Table 1:** Patient demographics.

Parameter	Value
Mean age in years (SD)	64.43 (10.02)
Gender (M/F)	108/107
Mean BMI in kg/m^2^ (SD)	29.03 (3.97)
BMI < 20	1
20 < BMI < 25	33
25 < BMI < 30	87
30 < BMI < 35	82
35 < BMI < 40	9
BMI > 40	3
AVN according to Steinberg	
0/I	8
II	10
III	35
IV	71
V	91

**Table 2:** Agreement between the planned prosthetic size and the actual size used.

Agreement	Femoral Stem		Acetabular Cup		Stem Neck Length AP X-ray
	AP X-ray	LL X-ray	AP X-ray	LL X-ray	
Exact match	167 (77.67%)	144 (66.98%)	157 (73.02%)	160 (74.42%)	162 (75.35%)
Predicted +1	20 (9.30%)	28 (13.02%)	25 (11.63%)	33 (15.35%)	7 (3.26%)
Predicted −1	19 (8.84%)	34 (15.81%)	22 (10.23%)	16 (7.44%)	32 (14.88%)
Predicted +2	3 (1.40%)	7 (3.26%)	7 (3.26%)	3 (1.40%)	1 (0.47%)
Predicted −2	5 (2.33%)	1 (0.47%)	1 (0.47%)	0 (0.00%)	13 (6.05%)
Predicted +3	0 (0.00%)	0 (0.00%)	2 (0.93%)	3 (1.40%)	0 (0.00%)
Predicted −3	1 (0.47%)	1 (0.47%)	0 (0.00%)	0 (0.00%)	0 (0.00%)
Predicted +4	0 (0.00%)	0 (0.00%)	1 (0.47%)	0 (0.00%)	0 (0.00%)

Results are expressed as the number of cases (percentages of total cases).

**Table 3:** Agreement rates between AP and LL projections for the planning of the femoral and acetabular components.

Agreement Rate between AP and LL Projections	Femoral Stem	Acetabular Cup
Complete correct agreement	126 (58.60%)	129 (60.00%)
LL overestimated by 1 or more sizes	19 (8.84%)	11 (5.12%)
LL underestimated by 1 or more sizes	26 (12.09%)	18 (8.37%)
AP overestimated by 1 or more sizes	12 (5.58%)	17 (7.91%)
AP underestimated by 1 or more sizes	10 (4.65%)	15 (6.98%)

Results are expressed as the number of cases (percentages of total cases).

## Data Availability

The data presented in this study are available on reasonable request from the corresponding authors.

## References

[1] Ethgen O., Bruyère O., Richy F., Dardennes C., Reginster J.Y. (2004). Health-related quality of life in total hip and total knee arthroplasty. A qualitative and systematic review of the literature. J. Bone Jt. Surg..

[2] Masaracchio M., Hanney W.J., Liu X., Kolber M., Kirker K. (2017). Timing of rehabilitation on length of stay and cost in patients with hip or knee joint arthroplasty: A systematic review with meta-analysis. PLoS ONE.

[3] Stoica C.I., Nedelea G., Cotor D.C., Gherghe M., Georgescu D.E., Dragosloveanu C., Dragosloveanu S. (2022). The Outcome of Total Knee Arthroplasty for Patients with Psychiatric Disorders: A Single-Center Retrospective Study. Medicina.

[4] Della Valle A.G., Padgett D.E., Salvati E.A. (2005). Preoperative planning for primary total hip arthroplasty. J. Am. Acad. Orthop. Surg..

[5] González Della Valle A., Slullitel G., Piccaluga F., Salvati E.A. (2005). The precision and usefulness of preoperative planning for cemented and hybrid primary total hip arthroplasty. J. Arthroplast..

[6] Ahmad S.S., Weinrich L., Giebel G.M., Beyer M.R., Stöckle U., Konrads C. (2021). Frontal knee alignment influences the vertical orientation of the femoral neck in standing position. Bone Jt. Open.

[7] Capello W.N. (1986). Preoperative planning of total hip arthroplasty. Instr. Course Lect..

[8] Ramme A.J., Fisher N.D., Egol J., Chang G., Vigdorchik J.M. (2018). Scaling Marker Position Determines the Accuracy of Digital Templating for Total Hip Arthroplasty. HSS J..

[9] Meyer C., Kotecha A., Richards O., Isbister E. (2009). Acetate templating for total hip arthroplasty using PACS. Ann. R. Coll. Surg. Engl..

[10] Iorio R., Siegel J., Specht L.M., Tilzey J.F., Hartman A., Healy W.L. (2009). A comparison of acetate vs digital templating for preoperative planning of total hip arthroplasty: Is digital templating accurate and safe?. J. Arthroplast..

[11] Bishi H., Smith J.B.V., Asopa V., Field R.E., Wang C., Sochart D.H. (2022). Comparison of the accuracy of 2D and 3D templating methods for planning primary total hip replacement: A systematic review and meta-analysis. EFORT Open Rev..

[12] Reinbacher P., Smolle M.A., Friesenbichler J., Draschl A., Leithner A., Maurer-Ertl W. (2022). Pre-operative templating in THA using a short stem system: Precision and accuracy of 2D versus 3D planning method. J. Orthop. Traumatol..

[13] Schiffner E., Latz D., Jungbluth P., Grassmann J.P., Tanner S., Karbowski A., Windolf J., Schneppendahl J. (2019). Is computerised 3D templating more accurate than 2D templating to predict size of components in primary total hip arthroplasty?. Hip Int..

[14] Sariali E., Mauprivez R., Khiami F., Pascal-Mousselard H., Catonné Y. (2012). Accuracy of the preoperative planning for cementless total hip arthroplasty. A randomised comparison between three-dimensional computerised planning and conventional templating. Orthop. Traumatol. Surg. Res..

[15] Inoue D., Kabata T., Maeda T., Kajino Y., Fujita K., Hasegawa K., Yamamoto T., Tsuchiya H. (2015). Value of computed tomography-based three-dimensional surgical preoperative planning software in total hip arthroplasty with developmental dysplasia of the hip. J. Orthop. Sci..

[16] Osmani F.A., Thakkar S., Ramme A., Elbuluk A., Wojack P., Vigdorchik J.M. (2017). Variance in predicted cup size by 2-dimensional vs 3-dimensional computerized tomography-based templating in primary total hip arthroplasty. Arthroplast. Today.

[17] Kosashvili Y., Shasha N., Olschewski E., Safir O., White L., Gross A., Backstein D. (2009). Digital versus conventional templating techniques in preoperative planning for total hip arthroplasty. Can. J. Surg..

[18] Bayraktar V., Weber M., von Kunow F., Zeman F., Craiovan B., Renkawitz T., Grifka J., Woerner M. (2017). Accuracy of measuring acetabular cup position after total hip arthroplasty: Comparison between a radiographic planning software and three-dimensional computed tomography. Int. Orthop..

[19] Petretta R., Strelzow J., Ohly N.E., Misur P., Masri B.A. (2015). Acetate templating on digital images is more accurate than computer-based templating for total hip arthroplasty. Clin. Orthop. Relat. Res..

[20] Blackley H.R., Howell G.E., Rorabeck C.H. (2000). Planning and management of the difficult primary hip replacement: Preoperative planning and technical considerations. Instr. Course Lect..

[21] Eggli S., Pisan M., Müller M.E. (1998). The value of preoperative planning for total hip arthroplasty. J. Bone Jt. Surg..

[22] Schiffers N., Schkommodau E., Portheine F., Radermacher K., Staudte H.W. (2000). Planning and performance of orthopedic surgery with the help of individual templates. Orthopade.

[23] Steinberg M.E. (1988). Management of avascular necrosis of the femoral head—An overview. Instr. Course Lect..

[24] Nogler M., Randelli F., Macheras G.A., Thaler M. (2021). Hemiarthroplasty of the hip using the direct anterior approach. Oper. Orthop. Traumatol..

[25] Strøm N.J., Reikerås O. (2018). Templating in uncemented THA. On accuracy and postoperative leg length discrepancy. J. Orthop..

[26] Cech A., Kase M., Kobayashi H., Pagenstert G., Carrillon Y., O’Loughlin P.F., Aït-Si-Selmi T., Bothorel H., Bonnin M.P. (2020). Pre-operative planning in THA. Part III: Do implant size prediction and offset restoration influence functional outcomes after THA?. Arch. Orthop. Trauma Surg..

[27] Dammerer D., Keiler A., Herrnegger S., Putzer D., Strasser S., Liebensteiner M. (2022). Accuracy of digital templating of uncemented total hip arthroplasty at a certified arthroplasty center: A retrospective comparative study. Arch. Orthop. Trauma Surg..

[28] Adamczyk A., Laboudie P., Nessek H., Kim P.R., Gofton W.T., Feibel R., Grammatopoulos G. (2023). Accuracy of digital templating in uncemented primary total hip arthroplasty: Which factors are associated with accuracy of preoperative planning?. Hip Int..

[29] Pongkunakorn A., Udomluck P., Aksornthung C., Wangjiraphan N. (2022). Digital Templating of THA Using PACS and an iPhone or iPad is as Accurate as Commercial Digital Templating Software. Clin. Orthop. Relat. Res..

[30] Efe T., El Zayat B.F., Heyse T.J., Timmesfeld N., Fuchs-Winkelmann S., Schmitt J. (2011). Precision of preoperative digital templating in total hip arthroplasty. Acta Orthop. Belg..

[31] Debarge R., Lustig S., Neyret P., Ait Si Selmi T. (2008). Confrontation of the radiographic preoperative planning with the postoperative data for uncemented total hip arthroplasty. Revue de Chirurgie Orthopédique et Réparatrice de l’Appareil Moteur.

[32] Di Martino A., Rossomando V., Brunello M., D’Agostino C., Pederiva D., Frugiuele J., Pilla F., Faldini C. (2023). How to perform correct templating in total hip replacement. Musculoskelet. Surg..

[33] Inoue D., Kabata T., Maeda T., Kajino Y., Yamamoto T., Takagi T., Ohmori T., Tsuchiya H. (2016). The correlation between clinical radiological outcome and contact state of implant and femur using three-dimensional templating software in cementless total hip arthroplasty. Eur. J. Orthop. Surg. Traumatol..

[34] Holzer L.A., Scholler G., Wagner S., Friesenbichler J., Maurer-Ertl W., Leithner A. (2019). The accuracy of digital templating in uncemented total hip arthroplasty. Arch. Orthop. Trauma Surg..

[35] Hofmann A.A., Bolognesi M., Lahav A., Kurtin S. (2008). Minimizing leg-length inequality in total hip arthroplasty: Use of preoperative templating and an intraoperative x-ray. Am. J. Orthop..

[36] Wang X., Ran G., Chen X., Jia H., Liu Z., Sun C., Ma L., Hou Z. (2021). Obturator Oblique and Pubic Ramus Inlet Views Can Better Guide the Insertion of an Anterior Column Acetabular Screw. Orthop. Surg..

[37] Salem H.S., Marchand K.B., Ehiorobo J.O., Tarazi J.M., Matzko C.N., Sodhi N., Hepinstall M.S., Mont M.A. (2020). Benefits of CT Scanning for the Management of Hip Arthritis and Arthroplasty. Surg. Technol. Int..

[38] Kagiyama Y., Otomaru I., Takao M., Sugano N., Nakamoto M., Yokota F., Tomiyama N., Tada Y., Sato Y. (2016). CT-based automated planning of acetabular cup for total hip arthroplasty (THA) based on hybrid use of two statistical atlases. Int. J. Comput. Assist. Radiol. Surg..

[39] Knafo Y., Houfani F., Zaharia B., Egrise F., Clerc-Urmès I., Mainard D. (2019). Value of 3D Preoperative Planning for Primary Total Hip Arthroplasty Based on Biplanar Weightbearing Radiographs. BioMed Res. Int..

[40] Murphy M.P., Boubekri A.M., Myall J.J., Ralles S.J., Brown N.M. (2022). Demographic Data Reliably Predicts Total Hip Arthroplasty Component Size. J. Arthroplast..

[41] Mevorach D., Perets I., Greenberg A., Kandel L., Mattan Y., Liebergall M., Rivkin G. (2022). The impact of femoral bone quality on cementless total hip pre-operative templating. Int. Orthop..

[42] Ashkenazi I., Morgan S., Shaked O., Snir N., Gold A., Khoury A., Shemesh S., Warschawski Y. (2023). The effect of patient body mass index and sex on the magnification factor during pre-operative templating for total hip arthroplasty. SICOT-J.

[43] Blevins J.L., Rao V., Chiu Y.F., Lyman S., Westrich G.H. (2020). Predicting implant size in total knee arthroplasty using demographic variables. Bone Jt. J..

[44] Chen J.B., Diane A., Lyman S., Chiu Y.F., Blevins J.L., Westrich G.H. (2022). Predicting Implant Size in Total Hip Arthroplasty. Arthroplast. Today.

[45] Chen X., Liu X., Wang Y., Ma R., Zhu S., Li S., Li S., Dong X., Li H., Wang G. (2022). Development and Validation of an Artificial Intelligence Preoperative Planning System for Total Hip Arthroplasty. Front. Med..

[46] Mandolini M., Brunzini A., Facco G., Mazzoli A., Forcellese A., Gigante A. (2022). Comparison of Three 3D Segmentation Software Tools for Hip Surgical Planning. Sensors.

[47] Thirion T., Georis P., Szecel Z., Gillet P. (2020). Preoperative planning of Total Hip Arthroplasty. Must this essential part of the procedure be necessarily performed by the orthopedic surgeon? A prospective study about 100 Corail ® Hip System. Acta Orthop. Belg..

[48] Peng H.M., Feng B., Chen X., Wang Y.O., Bian Y.Y., Wang W., Weng X.S., Qian W.W. (2021). Usefulness of a Simple Preoperative Planning Technique using Plain X-rays for Direct Anterior Approach for Total Hip Arthroplasty. Orthop. Surg..

[49] Shichman I., Factor S., Shaked O., Morgan S., Amzallag N., Gold A., Snir N., Warschawski Y. (2020). Effects of surgeon experience and patient characteristics on accuracy of digital pre-operative planning in total hip arthroplasty. Int. Orthop..

[50] Jung S., Neuerburg C., Kappe T., Wernerus D., Reichel H., Bieger R. (2012). Validity of digital templating in total hip arthroplasty: Impact of stem design and planner’s experience. Zeitschrift für Orthopädie und Unfallchirurgie.

